# Fixed points of multivalued convex contractions with application

**DOI:** 10.1371/journal.pone.0321860

**Published:** 2025-05-12

**Authors:** Abdul Rahim Khan, Hamed H. Al-Sulami, Muhammad Rashid, Faiza Shabbir

**Affiliations:** 1 Department of Mathematics and Statistics, University of Southern Punjab, Multan, Pakistan; 2 Department of Mathematics, King Abdulaziz University, Jeddah, Saudi Arabia; University of Education, PAKISTAN

## Abstract

In this work, we establish fixed point outcomes for single- valued convex contraction type mappings in the context of a b-metric space. Some of the new results are extended for a multivalued convex contraction and an F-convex contraction. Thereby, an analogue of the famous Nadler’s fixed point theorem for a multivalued convex contraction mapping is obtained. The relation among various contractions is presented in a diagram for an insight in this area of investigations. We apply a special case of Theorem 2.11, to solve a nonlinear Fredholm integral equation for a Chatterjea convex contraction.

## 1 Preliminaries and introduction

The exploration of fixed point theory represents a fundamental and highly impactful area in modern mathematics. The main theme in this subject is the celebrated principle of Banach contraction, which asserts that there is a unique fixed point for each contraction mapping on a complete metric space. The proof of this principle hinges on the iterates of the contraction and the principle itself has applications in a wide range of fields like partial differential equations, integral equations, image processing, optimization and artificial intelligence. In view of paramount importance of this principle, Browder and Petryshyn [7], Istratescu [11] and Berinde [2] have introduced and studied new classes of mappings enjoying higher powers of the mapping; in particular, Istratescu has coined the term “convex contraction”.

We now set out to develop results for convex contraction type single-valued as well as multi-valued mappings in the context of b-metric spaces.

### 1.1 Single-valued mappings

**Definition 1.1:** Consider a nonempty set *G* and a mapping S : G→G. A point g∈G is referred to as a fixed point of *S* if it satisfies S(g)=g. The set consisting of all fixed points of the mapping *S* is represented by Fix(S).

**Definition 1.2:** Let (*G*, *d*) be a complete metric space. A map S : G→G is known as Istratescu convex contraction [11] if


$$d(S^{2}(g),S^{2}(h))\leq ad(S(g),S(h))+bd(g,h)\;\text{for any}\;g,h\in G$$

where *a* and *b* are constants which satisfy 0 < *a*, *b* < 1 and a+b<1.

**Remark 1.3:** ([17],Example 2.1) (i) If *b* = 0, the convex contraction condition reduces to the well-known Banach contraction condition:


d(S(g),S(h))≤ad(g,h)for allg,h∈G

subject to a change of notation.

(ii) If *a* = 0, the convex contraction condition reduces to the classial "asymptotic” contraction:


$$d(S^{2}(g),S^{2}(h))\leq bd(g,h),$$

which confirms the presence of a fixed point, even when the number 2 is replaced with any arbitrary integer *n*.

**Example 1.4:** [17] Let G=[0,1] be equipped with the usual metric of ℝ. Define S : [0,1]→[0,1] by


$$S(g)=\frac{g^{2}+1}{2},\;g\in [0,1].$$

Then *S* is not a Banach contraction, and Fix(S)={1}. But *S* is a convex contraction, because for g,h∈G, we have


$$\left|S^{2}g-S^{2}h|\leq \frac{1}{2}|Sg-Sh|+\frac{1}{4}|g-h\right|$$

with $a=\frac{1}{2}$ and $b=\frac{1}{4}$.

**Definition 1.5:** [16] A mapping S : G→G is defined as a generalized convex contraction if there exist a function α:G×G→[0,∞) and constants a,b∈[0,1) satisfying a+b<1, so that the subsequent condition is satisfied:

$$\alpha (g,h)d(S^{2}(g),S^{2}(h))\leq ad(S(g),S(h))+bd(g,h)\;\text{for every }\;g,h\in G.$$ (1)

If *a* = 0 and α(g,h)=1 in Definition 1.5, then it becomes asymptotic contraction condition of Remark 1.3.

**Definition 1.6:** [9] A continuous map S : G→G on a complete metric space (*G*,d) is referred to as a two-sided convex contraction if there are constants $a_{1},a_{2},b_{1},b_{2}\in (0,1)$ and the following inequality is satisfied:


$$d(S^{2}(g),S^{2}(h))$$

$$\leq a_{1}d(g,S(g))+a_{2}d(S(g),S^{2}(g)+b_{1}d(h,S(h)+b_{2}d(S(h),S^{2}(h))$$

for all g,h∈G and $a_{1}+a_{2}+b_{1}+b_{2}\;<\;1$.

**Definition 1.7.** [10] Let *S* be a mapping from a metric space *G* into itself. The set


$$O(S,g)=\{S^{n}g:n=0,1,2,\ldots \}$$

is referred to as the orbit of *S* starting at *g*. We say that *S* is *orbitally continuous* at a point z∈G if for every sequence $\left\{g_{n}\}\subset O(S,g\right)$, with g∈G, the condition


$$\lim_{n\to \infty }g_{n}=z\;⟹\;\lim_{n\to \infty }S(g_{n})=S(z).$$

It is important to note that every continuous self-map on a metric space is orbitally continuous, the converse does not necessarily hold [6].

**Definition 1.8:** [9] Let (*G*, *d*) denote a complete metric space. A continuous mapping S : G→G is termed a Chatterjea two-sided convex contraction if there are constants $a_{1},a_{2},b_{1},b_{2}\in (0,1)$ and the subsequent inequality is satisfied:


$$d(S^{2}(g),S^{2}(h))$$

$$\leq a_{1}d(g,S(h))+a_{2}d(S(h),S^{2}(h))+b_{1}d(h,S(g))+b_{2}d(S(g),S^{2}(g))$$

for any g,h∈G and $a_{1}+a_{2}+b_{1}+b_{2}\;<\;1$.

We now provide an example of a Chatterjea two-sided convex contraction.

**Example 1.9:** Let G=[0,1] with the metric d(g,h)=|g−h|. Define S : [0,1]→[0,1] by:


$$S(g)=\frac{3g+1}{4},\;g\in [0,1]$$

Let us calculate ${\text{S}}^{2}(\text{g})$:


$$S^{2}(g)=S(S(g))=S\left(\frac{3g+1}{4}\right)=\frac{3\left(\frac{3g+1}{4}\right)+1}{4}=\frac{9g+7}{16}$$

$$d(g,S(h))=\left|g-\frac{3h+1}{4}\right|$$

$$d(S(h),S^{2}(h))=\left|\frac{3h+1}{4}-\frac{9h+7}{16}\right|=\frac{3|h-1|}{16}$$

$$d(h,S(g))=\left|h-\frac{3g+1}{4}\right|$$

$$d(S(g),S^{2}(g))=\left|\frac{3g+1}{4}-\frac{9g+7}{16}\right|=\frac{3|g-1|}{16}$$

$$d(S^{2}(g),S^{2}(h))=\left|\frac{9g+7}{16}-\frac{9h+7}{16}\right|=\frac{9|g-h|}{16}.$$

$$\frac{9|g-h|}{16}\leq \frac{1}{4}\left|g-\frac{3h+1}{4}\right|+\frac{1}{8}\cdot \frac{3|h-1|}{16}+\frac{1}{4}\left|h-\frac{3g+1}{4}\right|+\frac{1}{8}\cdot \frac{3|g-1|}{16}$$

where


$$a_{1}=\frac{1}{4},\;a_{2}=\frac{1}{8},\;b_{1}=\frac{1}{4},\;b_{2}=\frac{1}{8}$$

Since the sum of the coefficients is:


$$a_{1}+a_{2}+b_{1}+b_{2}=\frac{1}{4}+\frac{1}{8}+\frac{1}{4}+\frac{1}{8}=\frac{3}{4}\;<\;1,$$

therefore, *S* is a Chatterjea two-sided convex contraction.

**Definition 1.10:** [9] A mapping S : G→G (complete metric space) is called Hardy and Rogers convex contraction if there exist positive integers $a_{1},a_{2},b_{1},b_{2},c_{1},c_{2},e_{1},e_{2},f_{1},f_{2}\in (0,1)$ and the subsequent inequality is satisfied:


$$d(S^{2}(g),S^{2}(h))$$

$$\leq a_{1}d(g,h)+a_{2}d(S(g),S(h))+b_{1}d(g,S(g))+b_{2}d(S(g),S^{2}(g))+$$

$$c_{1}d(h,S(h))+c_{2}d(S(h),S^{2}(h))+e_{1}d(g,S(h)+e_{2}d(S(h),S^{2}(h))$$

$$+f_{1}d(h,S(g))+f_{2}d(S(g),S^{2}(g))$$

for any g,h∈G and $a_{1}+a_{2}+b_{1}+b_{2}+c_{1}+c_{2}+e_{1}+e_{2}+f_{1}+f_{2}\;<\;1$.

It is remarked that some of the above mentioned classes of convex contraction type mappings are independent of each other [9, 11]. In case, the constants are allowed to be zero in Definition 1.10, then it reduces to Istratescu convex contraction (Definition 1.2).

**Definition 1.11:** [16] Let S : G→G (metric space). For any given ϵ>0, a point $g_{0}\in G$ is called an *approximate fixed point* of *S* if it fulfills the condition:


$$d(g_{0},Sg_{0})\;<\;\epsilon .$$

**Definition 1.12:** [7] A map *S* defined on a metric space (*G*, *d*) is termed asymptotically regular at any point g∈G if


$$d(S^{n}(g),S^{n+1}(g))\to 0\text{ as }n\to \infty ,$$

where ${\text{S}}^{\text{n}}(\text{g})$ denotes the *n*-th iterate of *S* at *g*.

**Lemma 1.13:** ([16],Lemma 2.1) Suppose that (*G*, *d*) is a metric space and *S* is an asymptotically regular mapping on *G*. Then *S* has an approximate fixed point.

**Definition 1.14:** [14] Consider the mapping $F\;:\;{\mathbb{R}}^{+}\to \mathbb{R}$ that adheres to the subsequent properties:

(a) The function *F* is strictly increasing.
(b) A sequence $\{\alpha_{n}\}\subset {\mathbb{R}}^{+}$ of positive real numbers satisfies $\lim_{n\to \infty }\alpha_{n}=0$ if and only if $\lim_{n\to \infty }F(\alpha_{n})=-\infty$.
(c) If there exists a constant k∈(0,1), then $\lim_{n\to \infty }(\alpha_{n}^{k})F(\alpha_{n})=0$.

The class of functions *F* that satisfies conditions (a)-(c) is represented by ℱ.

**Definition 1.15:** An *F*-contraction is a self-map *S* defined over a metric space *G* if there is a function F∈ℱ and a constant τ>0 such that


τ+F(d(Sg,Sh))≤F((g,h)),

for all g,h∈G with *d*(*Sg*, *Sh*) > 0.

**Definition 1.16:** [26] Let *F* be a mapping that meets requirements (a)–(c). A funtion S : G→G is known as an *F*-Kannan mapping if the following hold:

(K1) Sg≠Sh⟹Sg≠g or Sh≠h
(K2) ∃γ>0 such that
  $$\gamma +F(d(Sg,Sh))\leq F\left(\frac{d(g,Sg)+d(h,Sh)}{2}\right)$$
  for all g,h∈G, with Sg≠Sh.

**Example 1.17:** ([26],Lemma 12) Consider a metric space (*G*, *d*) and F-Kannan mapping S : G→G. Then,


$$d(S^{n}(g),S^{n+1}(g))\to 0\;\text{as}\;n\to \infty \;\text{for all}\;g\in G.$$

So an F-Kannan mapping is asymptotically regular.

Here are some well known results for convex contraction type mappings.

**Theorem 1.18:** ([11], Theorem 1.2) Every convex contraction mapping defined on a complete metric space has a unique fixed point.

**Theorem 1.19:** ([5], Theorem 2.1) If *S* is a self-map on a complete metric space (*G*, *d*), g,h∈G, $a_{1},a_{2},b_{1},b_{2},c_{1},c_{2}\in (0,1)$ and $0\leq a_{1}+a_{2}+b_{1}+b_{2}+c_{1}+c_{2}\;<\;1,$ then we have


$$d(S^{2}(g),S^{2}(h))$$

$$\leq a_{1}d(g,h)+a_{2}d(S(g)),S(h))+b_{1}d(g,S(g))+b_{2}d(S(g),S^{2}(g))$$

$$+c_{1}d(h,S(h))+c_{2}d(S(h),S^{2}(h)).$$

Suppose that *S* is *k*-continuous for k∈ℕ [12]. Then *S* admits a unique fixed point.

**Theorem 1.20:** ([6],Theorem 2.1) Let *S* be a self-map of a complete metric space (*G*, *d*) such that for each g,h∈G;


$$d(S^{m}(g),S^{m}(h))\leq a_{j}d(S^{j}(g),S^{j}(h)),$$

where the constants $a_{0},a_{1},\ldots ,a_{m-1}$ are non-negative and their sum satisfies $\sum_{j=0}^{m-1}a_{j}\;<\;1$. The map *S* has a unique fixed point if it is either orbitally continuous or k-continuous.

**Theorem 1.21:** ([9], Theorem 2.5) Let (*G*, *d*) be a complete metric space and *S* be a self-map on *G* satisfying the condition:


$$d(S^{2}(g),S^{2}(h))$$

$$\leq a_{1}d(g,S(h))+a_{2}d(S(h),S^{2}(h))+b_{1}d(h,S(g))+b_{2}(S(g),S^{2}(g)),$$

for all g,h∈G, where $a_{1},a_{2},b_{1},b_{2}\in (0,1)$ and $a_{1}+a_{2}+b_{1}+b_{2}\;<\;1$. If *S* is orbitally continuous, then *S* admits a unique fixed point in *G*. Moreover, for any initial point $g_{0}\in G$, the Picard iterations sequence $\{g_{n}{\}}_{n=0}^{\infty }$, defined by $g_{n+1}=S(g_{n})$ for n≥0, converges to a fixed point of *S* in *G*.

### 1.2 Multivalued mappings

**Definition 1.22:** Let (*G*, *d*) be a metric space and *CB*(*G*) represent the family of closed and bounded subsets of *G*. A mapping S : G→CB(G) has *g*_0_ as it’s fixed point if $g_{0}\in Sg_{0}$.

**Definition 1.23** [12] A map *S* on a metric space (*G*, *d*) is known as asymptotically regular at a point g∈G if


$$H(S^{n}(g),S^{n+1}(g))\to 0\;as\;n\to \infty \text{,}$$

*H* is the Hausdorff metric given by

H(A,B)=max{sup{d(a,B):a∈A},sup{d(b,A):b∈B}}

where d(a,B)=inf{d(a,b):b∈B}.

**Definition 1.24:** Consider the metric space (*G*, *d*). A mapping S : G→CB(G) is called convex contraction if


$$H(S^{2}(g),S^{2}(h))\leq ad(S(g),S(h))+bd(g,h)$$

for all g,h∈G, where *a*, *b* are the constants that fulfill 0 < *a*, *b* < 1 and a+b<1.

**Definition 1.25:** Let (*G*, *d*) be a metric space. A mapping S : G→CB(G) is called Chatterjea two-sided convex contraction if the following holds:


$$H(S^{2}(g),S^{2}(h))$$

$$\leq a_{1}d(g,S(h))+a_{2}d(S(h),S^{2}(h))+b_{1}d(h,S(g))+b_{2}d(S(g),S^{2}(g)),$$

for every g,h∈G, $a_{1},a_{2},b_{1},b_{2}\in (0,1)$ and $a_{1}+a_{2}+b_{1}+b_{2}\;<\;1$.

**Definition 1.26:** Let (*G*, *d*) be a complete metric space and S : G→CB(G) be a mapping. The mapping *S* is said to be a weak convex contraction if it satisfies:


$$H(S^{2}(g),S^{2}(h))$$

$$\leq a_{0}d(g,h)+b_{0}d(h,S(g))+a_{1}d(S(g),(Sh))+b_{1}d(S(h),S^{2}(g))$$

for all g,h∈G, $b_{0},b_{1}\geq 0$, $0\;<\;a_{0},a_{1}\;<\;1$ and $a_{0}+a_{1}\;<\;1.$

**Definition 1.27:** [2] Let (*G*, *d*) be a metric space and S:G→CB(G) be a multivalued map. If θ∈(0,1) and L≥0, then *S* is a multivalued weak contraction if


H(Sg,Sh)≤θd(g,h)+Ld(h,Sg),

holds for each pair of points g,h∈G.

For ease of reference, we provide proof of the result to follow.

**Lemma 1.28:** ([2], Lemma 1) Consider a metric space (*G*, *d*), two subsets A,B⊆G and a fixed constant *q* > 1. Then, for each a∈A, there exists an element b∈B such that


d(a,b)≤qH(A,B).

**Proof:** For H(A,B)=0, the result holds for *b* = *a* with a∈B.

Set $\epsilon =(k^{-1}-1)H(A,B)\;>\;0$ where *k* < 1.

According to the definitions of *d*(*a*, *B*) and *H*(*A*, *B*), for all ϵ>0, there exists an element b∈B such that


d(a,b)≤d(a,B)+ϵ≤H(A,B)+ϵ. (*)

Putting the selected value of ϵ in the above inequality, we get the result with *q* = *k*^−1^.

Fixed point results for single-valued asymptotically regular and convex contraction type mappings have been considered by Berinde and Pacurar [3], Bisht and Hussain [4] and Khan and Oyetunbi [12] while Khan and Oyetunbi [13], Karakaya and Sekman [15] and Sgroi and Vetro [23] have studied these results for multivalued mappings.In this paper, we will extend Theorem 1.21 for a Chatterjea two-sided convex contraction in a b-metric space. On the one hand, we apply our new result to solve a non-linear Fredholm integral equation and on the other hand, we find it’s multivalued version. A fixed point result for generalized convex contraction on a b-metric space is proved in Theorem 2.5. We established a multivalued version of Theorem 1.18, the fundamental result of Istratescu for a convex contraction.

Recently, Nallaselli *et al* [19], Özkan *et al* [21] and Ricinschi *et al* [22] have studied convex contractions on metric spaces and b-metric spaces. It is remarked that our results and techniques are different from their ones and are more focused on the development of fixed point results for a multivalued convex contraction.

## 2 Fixed points results

### 2.1 Single-valued mappings

The concept of a b-metric space was presented by Czerwik [8] as follows:

**Definition 2.1:** Let *G* be a nonempty set and s≥1 be a fixed real number. A function $d:G\times G\to \;{\mathbb{R}}^{+}$ is said to be a b-metric if, for all g,h,z∈G, the subsequent conditions hold:

(b_1_) d(g,h)=0, ⟺ *g* = *h*
(b_2_) d(g,h)=d(h,g)
(b_3_) d(g,h)≤s[d(g,z)+d(z,h)].

The triplet (*G*, *d*, *s*) is then referred to as a b-metric space.

Note that when *s* = 1, a b-metric space becomes a metric space. But, in general, the converse does not hold.

We extend Theorem 2.5 in [9] for b-metric spaces as follows.

**Theorem 2.2:** Let (*G*, *d*, *s*) be a complete b-metric space with coefficient s≥1 and *S* be a self-map on *G* satisfying the condition;


$$d(S^{2}(g),S^{2}(h))$$

$$\leq a_{1}d(g,S(h))+a_{2}d(S(h),S^{2}(h))+b_{1}d(h,S(g))+b_{2}d(S(g),S^{2}(g)),$$ (2)

for all g,h∈G, $a_{1},a_{2},b_{1},b_{2}\in (0,1)$, $a_{1}+a_{2}+b_{1}+b_{2}\;<\;1$ and $2sa_{1}+b_{2}+a_{2}\;<\;1$.

If *S* is orbitally continuous, then it admits a unique fixed point in *G*. For any initial point $g_{0}\in G$, the sequence $\{g_{n}{\}}_{n=0}^{\infty }$, defined recursively by $g_{n+1}=Sg_{n}$ for n≥0, converges to the unique fixed point of *S* in *G*.

**Proof:** Let $g_{0}\in G$ be an arbitrary point. Define a sequence {*g*_*n*_} by


$$g_{1}=S(g_{0}),$$

$$g_{2}=S(g_{1}),$$

$$g_{3}=S(g_{2}),$$

⋯

$$g_{n+1}=S(g_{n})=S^{n}(g_{0}),$$

for all n=0,1,2,….

Put *g* = *g*_0_, *h* = *S*(*g*_0_) in (2), we get


$$d(S^{2}(g_{0}),S^{3}(g_{0}))$$

$$\leq a_{1}d(g_{0},S^{2}(g_{0}))+a_{2}d(S^{2}(g_{0}),S^{3}(g_{0}))+b_{1}d(S(g_{0}),S(g_{0}))+$$

$$b_{2}d(S(g_{0}),S^{2}(g_{0}))$$

$$\leq a_{1}d(g_{0},S^{2}(g_{0}))+a_{2}d(S^{2}(g_{0}),S^{3}(g_{0}))+b_{2}d(S(g_{0}),S^{2}(g_{0}))$$

$$\leq a_{1}s[d(g_{0},S(g_{0}))+d(S(g_{0}),S^{2}(g_{0}))]+a_{2}d(S^{2}(g_{0}),S^{3}(g_{0}))+$$

$$b_{2}d(S(g_{0}),S^{2}(g_{0}))$$

$$\leq sa_{1}d(g_{0},S(g_{0}))+sa_{1}d(S(g_{0}),S^{2}(g_{0}))+a_{2}d(S^{2}(g_{0}),S^{3}(g_{0}))+$$

$$b_{2}d(S(g_{0}),S^{2}(g_{0}))$$

$$\leq sa_{1}d(g_{0},S(g_{0}))+(sa_{1}+b_{2})d(S(g_{0}),S^{2}(g_{0}))+a_{2}d(S^{2}(g_{0}),S^{3}(g_{0}))$$

$$\leq (2\;sa_{1}+b_{2})\max\{d(x_{0},S(g_{0})),d(S(g_{0}),S^{2}(g_{0}))\}+a_{2}d(S^{2}(g_{0}),S^{3}(g_{0})).$$

$$\leq \frac{\left(2\;sa_{1}+b_{2}\right)}{1-a_{2}}\max\{d(g_{0},S(g_{0})),d(S(g_{0}),S^{2}(g_{0}))\},$$

$$\leq \frac{2\;sa_{1}+b_{2}}{1-a_{2}}k\;\text{where}\;k=\max\{d(g_{0},S(g_{0})),d(S(g_{0}),S^{2}(g_{0}))\}.$$

Now substitute *g* = *S*(*g*_0_) and $h=S^{2}(g_{0})$ in (2), and get:


$$d(S^{3}(g_{0}),S^{4}(g_{0}))$$

$$\leq a_{1}d(S(g_{0}),S^{3}(g_{0}))+a_{2}d(S^{3}(g_{0}),S^{4}(g_{0}))+b_{1}d(S^{2}(g_{0}),S^{2}(g_{0}))+$$

$$b_{2}d(S^{2}(g_{0}),S^{3}(g_{0}))$$

$$\leq a_{1}d(S(g_{0}),S^{3}(g_{0}))+a_{2}d(S^{3}(g_{0}),S^{4}(g_{0}))+b_{2}d(S^{2}(g_{0}),S^{3}(g_{0}))$$

$$\leq sa_{1}[d(S(g_{0}),S^{2}(g_{0}))+d(S^{2}(g_{0}),S^{3}(g_{0}))]+a_{2}d(S^{3}(g_{0}),S^{4}(g_{0}))+$$

$$b_{2}d(S^{2}(g_{0}),S^{3}(g_{0}))$$

$$\leq sa_{1}d(S(g_{0}),S^{2}(g_{0}))+sa_{1}d(S^{2}(g_{0}),S^{3}(g_{0}))+a_{2}d(S^{3}(g_{0}),S^{4}(g_{0}))+$$

$$b_{2}d(S^{2}(g_{0}),S^{3}(g_{0}))$$

$$\leq \frac{2\;sa_{1}+b_{2}}{1-a_{2}}k.$$

By continuing this process, we obtain:


$$d(S^{n}(g_{0}),S^{n+1}(g_{0}))\leq {\left(\frac{2\;sa_{1}+b_{2}}{1-a_{2}}\right)}^{n-2}k$$

$$⟹d(S^{n}(g_{0}),S^{n+1}(g_{0}))\leq \gamma^{n-2}k,\;\text{where}\;\gamma =\frac{2\;sa_{1}+b_{2}}{1-a_{2}}\;<\;1.$$

We now demonstrate that {*g*_*n*_} forms a Cauchy sequence in *G*. For *m* < *n*, we get


$$d(S^{n}(g_{0}),S^{m}(g_{0}))$$

$$\leq s[d(S^{n}(g_{0}),S^{n+1}(g_{0}))+d(S^{n+1}(g_{0}),S^{m}(g_{0}))]$$

$$\leq sd(S^{n}(g_{0}),S^{n+1}(g_{0}))+sd(S^{n+1}(g_{0}),S^{m}(g_{0}))$$

$$\leq sd(S^{n}(g_{0}),S^{n+1}(g_{0}))+s^{2}d(S^{n+1}(g_{0}),S^{n+2}(g_{0}))+$$

$$s^{3}d(S^{n+2}(g_{0}),S^{n+3}(g_{0}))+\ldots +s^{\left(m-1\right)}d(S^{m-1}(g_{0}),S^{m}(g_{0}))$$

$$\leq s\gamma^{\left(n-2\right)}k+s^{2}\gamma^{\left(n-1\right)}k+s^{3}\gamma^{n}k+\ldots$$

$$\leq s\gamma^{\left(n-2\right)}[1+s\gamma +s^{2}\gamma^{2}+\ldots ]k$$

$$\leq \frac{s\gamma^{\left(n-2\right)}}{1-s\gamma }k.$$

$$⟹d(S^{n}(g_{0}),S^{m}(g_{0}))\leq \frac{s\gamma^{\left(n-2\right)}}{1-s\gamma }k$$

As n→∞, in the view of sγ<1, $d(S^{n}(g_{0}),S^{m}(g_{0}))\to 0$.

Therefore $d(S^{n}(g_{0}),S^{n+1}(g_{0}))$ is a Cauchy sequence. Since *G* is complete, there exists a point g∈G such that $g_{n}\to g$ as n→∞. So applying orbital continuity of *S*, we get


$$\lim_{n\to \infty }S^{\left(n+1\right)}(g_{0})=S(g).$$

This shows that *g* is a fixed point of *S*.

For it’s uniqueness, assume on the contrary that *u* is another fixed point of *S* such that g ≠ u.


$$d(g,u)=d(S^{2}(g),S^{2}(u))\leq a_{1}d(g,S(u)+a_{2}d(S(u),S^{2}(u))+b_{1}d(u,S(g))+$$

$$b_{2}d(S(g),S^{2}(g))$$

$$\leq a_{1}d(g,S(u)+a_{2}d(S(u),S(u))+b_{1}d(u,S(g))+b_{2}d(S(g),S(g))$$

$$\leq a_{1}d(g,S(u)+b_{1}d(u,S(g))$$

$$\leq a_{1}d(g,u)+b_{1}d(u,g)$$

$$\leq (a_{1}+b_{1})d(g,u).$$

$$⟹d(g,u)-(a_{1}+b_{1})d(g,u)\leq 0$$

$$(1-a_{1}-b_{1})d(g,u)\leq 0$$

As   $1-a_{1}-b_{1}\;<\;0$, so d(g,u)=0 implies *g* = *u* which is a contradiction. Hence *S* has a unique fixed point in *G*.

An extension of Corollary 1 in [1] for b-metric spaces is presented below.

**Corollary 2.3:** Let *S* be a self-map on a complete b-metric space (G,d,s) satisfying (2) in Theorem 2.2 with $a_{1}+a_{2}+b_{1}+b_{2}\;<\;1$. Then both *S* and *S*^2^ have a unique common fixed point (i.e. *S*(*z*) = *S*^2^(*z*) = *z*).

**Proof:** By Theorem 2.2, there exists a unique fixed point *z* of *S* and so z∈Fix(S).

Now, we aim to show that *S* and *S*^2^ have a unique common fixed point. Clearly, $F(S)\subseteq F(S^{2})$. Suppose $u\in F(S^{2})$ and u≠z. Then *S*^2^(*u*) = *u*.

Assume, for a contradiction, that S(u)≠u. Since *S*^2^(*u*) = *u*, therefore by (2), applied to the pair *u* and S(u),we get


$$d(S(u),u)=d(S^{2}(S(u)),S^{2}(u)),$$

$$d(S(u),u)\leq a_{1}d(u,S(u))+a_{2}d(S(u),S^{2}(u))+b_{1}d(S(u),u)+b_{2}d(u,S(u)).$$

$$d(S(u),u)\leq a_{1}d(u,S(u))+a_{2}d(S(u),u)+b_{1}d(S(u),u)+b_{2}d(u,S(u)),$$

$$d(S(u),u)\leq (a_{1}+b_{2})d(u,S(u))+(a_{2}+b_{1})d(S(u),u).$$

Now, if d(S(u),u)≠0, the above inequality implies that d(S(u),u) is smaller than itself, which is a contradiction. Thus, S(u)=u, and hence u∈Fix(S). Therefore, *F*(*S*) = *F*(*S*^2^), and since *z* is the only fixed point of *S*, it must also be the only fixed point of *S*^2^. This shows that both *S* and *S*^2^ possess a unique common fixed point.

**Definition 2.4** [16] Consider a self-map *S* defined on a nonempty set *G*, along with a function α : G×G→[0,∞). *S* is α-admissible if for g,h∈G,


α(g,h)≥1⟹α(Sg,Sh)≥1.

We now extend ([16],Theorem 3.1) in the framework of b-metric spaces.

**Theorem 2.5:** Consider a *b*-metric space (*G*, *d*, *s*) with s≥1, *S* a generalized convex contraction on *G* characterized by the base mapping α. Assume that *S* satisfies the α-admissibility condition and that there exists a point $g_{0}\in G$ such that $\alpha (g_{0},S(g_{0}))\geq 1$. Under these assumptions, *S* possesses an approximate fixed point. Furthermore, if *S* is continuous and (*G*, *d*, *s*) is complete, then *S* has a fixed point.

**Proof:** Let $g_{0}\in G$ be such that $\alpha (g_{0},S(g_{0}))\geq 1$. Define {*g*_*n*_} in *G* as before to get


$$g_{n+1}=S(g_{n})=S^{n}(g_{0})\;\text{for all }n\geq 0.$$

If $g_{n}=g_{n+1}$ for some *n*, the proof is complete. Otherwise, assume $g_{n}\neq g_{n+1}$. Given that *S* is α-admissible, we have the condition $\alpha (g_{0},S(g_{0}))\geq 1$ for all *n*. Let $v=d(S(g_{0}),S^{2}(g_{0}))+\;d(g_{0},S(g_{0}))$ and λ=a+b.

Then $d(S(g_{0}),S^{2}(g_{0}))\leq v$.

Now put *g* = *g*_0_, *h* = *S*(*g*_0_) in (1) and get


$$d(S^{2}(g_{0}),S^{3}(g_{0}))\leq \alpha (S(g_{0}),g_{0})\;d(S^{2}(g_{0}),S^{3}(g_{0}))$$

$$\leq a\;d(S(g_{0}),S^{2}(g_{0}))+b\;d(g_{0},S(g_{0}))$$

≤λv

Again put *g* = *Sg*_0_ and $h=S^{2}g_{0}$ in (1) to obtain.


$$d(S^{3}(g_{0}),S^{4}(g_{0}))\leq \alpha (S(g_{0}),S^{2}(g_{0}))d(S^{3}(g_{0}),S^{4}(g_{0}))$$

$$\leq ad(S^{2}(g_{0}),S^{3}(g_{0}))+bd(S(g_{0}),S^{2}(g_{0}))$$

≤λv

Similarly,


$$d(S^{4}(g_{0}),S^{5}(g_{0}))\leq \lambda^{2}v$$

And also,


$$d(S^{5}(g_{0}),S^{6}(g_{0}))\leq \lambda^{2}v$$

By continuing this procedure, we get


$$d(S^{m}(g_{0}),S^{m+1}(g_{0}))\leq \lambda^{l}v$$

where *m* = 2*l* or *m* = 2*l*  +  1. Consequently, $d(S^{m}g_{0},S^{m+1}g_{0})\to 0$ as n→∞. This implies that *S* is asymptotically regular. By Lemma 1.13, *S* possesses an approximate fixed point. Now, suppose that *S* is continuous, and the space (*G*, *d*) is a complete *b*-metric space. To demonstrate that {*g*_*n*_} is a Cauchy sequence, consider *m* and *n* as positive integers such that *m* < *n*. Let *m* = 2*l* and *n* = 2*p*, where l≥1 and p≥2.


$$d(S^{m}(g_{0}),S^{n}(g_{0}))$$

$$\leq s[d(S^{m}(g_{0}),S^{m+1}(g_{0}))+d(S^{m+1}(g_{0}),S^{n}(g_{0}))]$$

$$\leq sd(S^{m}(g_{0}),S^{m+1}(g_{0}))+sd(S^{m+1}(g_{0}),S^{n}(g_{0}))$$

$$\leq sd(S^{m}(g_{0}),S^{m+1}(g_{0}))+s[d(S^{m+1}(g_{0}),S^{m+2}(g_{0}))+d(S^{m+2}(g_{0}),S^{n}(g_{0}))]$$

$$\leq sd(S^{m}(g_{0}),S^{m+1}(g_{0}))+s^{2}d(S^{m+1}(g_{0}),S^{m+2}(g_{0}))+s^{2}d(S^{m+2}(g_{0}),S^{n}(g_{0}))$$

$$\leq sd(S^{m}(g_{0}),S^{m+1}(g_{0}))+s^{2}d(S^{m+1}(g_{0}),S^{m+2}(g_{0}))+\ldots +s^{n-1}$$

$$d(S^{n-1}(g_{0}),S^{n}(g_{0}))$$

$$=sd(S^{2l}(g_{0}),S^{2l+1}(g_{0}))+s^{2}d(S^{2l+1}(g_{0}),S^{2l+2}(g_{0}))+\ldots +s^{2p-1}$$

$$d(S^{2p-1}(g_{0}),S^{2p}(g_{0}))$$

$$\leq s\lambda^{l}v+s^{2}\lambda^{l}v+s^{3}\lambda^{l+1}v+\ldots +s^{2p-1}\lambda^{p-1}v$$

$$\leq \left(s\lambda^{l}v+s^{3}\lambda^{l+1}v+s^{5}\lambda^{l+2}+\cdots \right)+\left(s^{2}\lambda^{l}v+s^{4}\lambda^{l+1}v+s^{6}\lambda^{l+2}+\cdots \right)$$

$$\leq s\lambda^{l}(1+s^{2}\lambda +\cdots )v+s^{2}\lambda^{l}\left(1+s^{2}\lambda^{l}+\cdots \right)v$$

$$\leq (s+s^{2})\lambda^{l}\left(1+s^{2}\lambda +\cdots \right)v$$

$$\leq (s+s^{2})\lambda^{l}\frac{1}{1-s^{2}\lambda }v$$

For *m* = 2*l* + 1 and *n* = 2*p*, where p≥1 and l≥1, with the condition that m<n, we obtain:


$$d(S^{m}(g_{0}),S^{n}(g_{0}))$$

$$\leq sd(S^{m}(g_{0}),S^{m+1}(g_{0}))+s^{2}d(S^{m+1}(g_{0}),S^{m+2}(g_{0}))+\ldots +s^{n-1}$$

$$d(S^{n-1}(g_{0}),S^{n}g_{0})$$

$$\leq sd(S^{2l+1}(g_{0}),S^{2l+2}(g_{0}))+s^{2}d(S^{2l+2}(g_{0}),S^{2l+3}(g_{0}))+\ldots +s^{2p-1}$$

$$d(S^{2p-1}(g_{0}),S^{2p}(g_{0}))$$

$$\leq s\lambda^{l}v+s^{2}\lambda^{l}v+s^{3}\lambda^{l+1}v+\ldots$$

$$\leq (s\lambda^{l}v+s^{3}\lambda^{l+1}v+s^{5}\lambda^{l+2}\ldots )+(s^{2}\lambda^{l}v+s^{4}\lambda^{l+1}v+s^{6}\lambda^{l+2}\ldots )$$

$$\leq s\lambda^{l}(1+s^{2}\lambda +\ldots )v+s^{2}\lambda^{l}(1+s^{2}\lambda^{l}+\ldots )v$$

$$\leq (s+s^{2})\lambda^{l}\{1+s^{2}\lambda +\ldots \}v$$

$$\leq (s+s^{2})\lambda^{l}\left(\frac{1}{1-s^{2}\lambda }\right)v$$

Similarly, let *m* = 2*l* + 1 and *n* = 2*p* + 1, where p≥2 and l≥1.


$$d(S^{m}(g_{0}),S^{n}(g_{0}))\leq (s+s^{2})\lambda^{l}{\left(\frac{1}{1-s^{2}\lambda }\right)}^{v}$$

For l→∞, in all the cases, we obtain $d(S^{m}(g_{0}),S^{n}(g_{0}))\to 0$ (in view of $s^{2}\lambda \;<\;1$). That is, {*g*_*n*_} is a Cauchy sequence in *G*. As *G* is complete, so there exists z∈G such that $g_{n}=S^{n}g_{n}\to z\in G$ as n→∞. If *S* is continuous, then $Sg_{n}\to Sz$, i.e., $g_{n+1}\to Sz$. Hence *Sz* = *z*.

Now we need to prove that *S* has a unique fixed point in *G*. Assume, on the contrary that ${\text{z}}^{*}$ is a fixed point of *S* such that $z\neq z^{*}$.

Taking *z* = *g* and z^*^ = *h* in (1), we get by hypothesis $\alpha (z,z^{*})\geq 1,$


$$d(z,z^{*})=d(S^{2}z,S^{2}z^{*})$$

$$\leq \alpha (z,z^{*})d(S^{2}z,S^{2}z^{*})$$

$$\leq ad(Sz,Sz^{*})+bd(z,z^{*})$$

$$\leq ad(z,z^{*})+bd(z,z^{*})$$

$$\leq (a+b)d(z,z^{*}),$$

$$(1-a-b)d(z,z^{*})\leq 0.$$

In view of (1–*a*–*b*) < 0, *d*(*z*,z^*^) = 0.Hence *z* = z^*^, which is a contradiction. Hence *S* has a unique fixed point in *G*.

**Lemma 2.6:** ([18], Lemma 4) Let k∈ℕ and {*g*_*n*_} represent a sequence in a *b*-metric space (*G*, *d*, *s*) such that

$$d(g_{n+k},g_{n+k-1})\leq \sum_{i=0}^{k-1}a_{i}d(g_{n+i},g_{n+i-1})$$ (3)

for all n∈ℕ, where $a_{i}\geq 0$ and


$$\sum_{i=0}^{k-1}a_{i}\;<\;1.$$

Then {*g*_*n*_} is Cauchy.

The following result improves ([18],Theorem 1).

**Theorem 2.7:** Let S : G→G be a convex contraction of order *k* defined on a complete *b*-metric space (*G*, *d*, *s*), such that


$$d(S^{k}g,S^{k}h)\leq \sum_{i=0}^{k-1}a_{i}d(S^{i}g,S^{i}h)$$

for all g,h∈G, where *a*_*i*_ is non-negative and the sum satisfies $\sum_{i=0}^{k-1}a_{i}\;<\;1$. If *S* exhibits orbital continuity, then *S* admits a unique fixed point in *G*.

**Proof** Let $g_{0}\in G$ be arbitrary. Define a sequence {*g*_*n*_}, as before, to get


$$g_{n+1}=S(g_{n}).$$

Now,


$$d(g_{n+k},g_{n+k-1})=d(S^{k}(g_{n}),S^{k}(g_{n-1}))$$

$$\leq \sum_{i=0}^{k-1}a_{i}d(S^{i}(g_{n}),S^{i}(g_{n-1}))$$

$$\leq \sum_{i=0}^{k-1}a_{i}d(S^{i}(g_{n+i}),S^{i}(g_{n+i-1}))$$

for every n∈ℕ, where *a*_*i*_ is non-negative, and the sum $\sum_{i=0}^{k-1}a_{i}\;<\;1$.

By Lemma 2.6, the sequence {*g*_*n*_} forms a Cauchy sequence in the complete space *G*. Hence, there exists a point z∈G such that $g_{n}\to z$ as n→∞. Furthermore, since $S(g_{n})\to z$, the orbital continuity of *S* ensures that $\lim_{n\to \infty }S(g_{n})=S(z)$. Consequently, we have S(z)=z, implying that *z* is a fixed point of *S*, and uniqueness of *z* follows as before.

### 2.2 Multi-valued mappings

**Lemma 2.8:** [25] Let (*G*, *d*, *s*) be a *b*-metric space and CB(G) denotes the class of all nonempty, closed, and bounded subsets of *G*. For any U,V∈CB(G), the following are satisfied:

1.
d(a,U)≤H(U,V),a∈U;
2.
For ε>0 and a∈U, ∃b∈V such that d(a,b)≤H(U,V)+ε.

**Definition 2.9** (cf. [25],Definition 2.4) Let *G* be an arbitrary nonempty set and s≥1 be a fixed real number. A strong *b*-metric on *G* is a function d : G×G→ℝ, satisfying the following axioms for g,h,z∈G :

(a) d(g,h)≥0;
(b) d(g,h)=0⇔g=h;
(c) d(g,h)=d(h,g);
(d) d(g,h)≤d(g,z)+sd(z,h).

The triplet (G,d,s) denotes a strong *b*-metric space.

We establish a multivalued version of Theorem 1.18, a classical result of Istratescu [11].

**Theorem 2.10:** Let (*G*, *d*,*s*) be a complete strong *b*-metric space and S : G→CB(G) be an asymptotically regular convex contraction. Then there exists h∈G such that h∈Sh.

**Proof:** Let $g_{0}\in G$. Then $Sg_{0}\neq \phi$ is a closed and bounded subset of *G*. Furthermore, let $g_{1}\in Sg_{0}$ and $Sg_{1}\neq \phi$ be a closed and bounded subset of *G*. By Lemma 2.8 (2), there exists $g_{2}\in Sg_{1}$ such that

$$d(g_{1},g_{2})\leq H(S^{2}(g_{0}),S^{2}(g_{1}))+\alpha .$$ (4)

Using the definition of convex contraction and asymptotic regularity of *S*, we get


$$d(g_{1},g_{2})\leq \alpha d(g_{0},g_{1})+\beta d(S(g_{0}),S(g_{1}))+\alpha$$

$$\leq \alpha d(g_{0},g_{1})+\beta d(S(g_{0}),S^{2}g_{0}))+\alpha$$

$$d(g_{1},g_{2})\leq \alpha d(g_{0},g_{1})+\alpha .$$ (5)

Now, $Sg_{2}\neq \phi$ is a closed and bounded subset of *G*, so there exists $g_{3}\in Sg_{2}$ such that


$$d(g_{2},g_{3})\leq H(S^{2}(g_{1}),S^{2}(g_{2}))+\alpha^{2}.$$

As before,


$$d(g_{2},g_{3})\leq \alpha d(g_{1},g_{2})+\beta d(S(g_{1}),S(g_{2}))+\alpha^{2}$$

$$\leq \alpha d(g_{1},g_{2})+\beta d(S(g_{1}),S^{2}(g_{1}))+\alpha^{2}$$

$$d(g_{2},g_{3})\leq \alpha d(g_{1},g_{2})+\alpha^{2}.$$ (6)

Similarly,


$$d(g_{3},g_{4})\leq H(S^{2}(g_{2}),S^{2}(g_{3}))+\alpha^{3}$$

$$\leq \alpha d(g_{2},g_{3})+\alpha^{3}.$$ (7)

Using (6), we have


$$d(g_{3},g_{4})\leq \alpha (\alpha d(g_{1},g_{2})+\alpha^{2})+\alpha^{3}$$

$$d(g_{3},g_{4})\leq \alpha^{2}d(g_{1},g_{2})+2\alpha^{3}$$

$$\leq \alpha^{2}d(g_{1},g_{2})+2\alpha^{3}$$

$$d(g_{3},g_{4})\leq \alpha^{2}(\alpha d(g_{0},g_{1})+\alpha )+2\alpha^{3}$$

$$\leq \alpha^{3}d(g_{0},g_{1})+3\alpha^{3}.$$

In general,


$$d(g_{n},g_{n+1})\leq \alpha^{n}d(g_{0},g_{1})+n\alpha^{n}.$$

For convenience, we set


$$d(g_{n},g_{n+1})=d_{n},$$

so the above result can be written as

$$d_{n}\leq \alpha^{n}d_{0}+n\alpha^{n}.$$ (8)

For m,n∈ℕ, m≥n, we have


$$d(g_{n},g_{m})$$

$$\leq d(g_{n},g_{n+1})+sd(g_{n+1},g_{n+2})+s^{2}d(g_{n+2},g_{n+3})+\cdots +s^{m-n-1}d(g_{m-1},g_{m}).$$

Using (8), we get


$$d(g_{n},g_{m})\leq \alpha^{n}d_{0}+s\alpha^{n+1}d_{0}+s^{2}\alpha^{n+2}d_{0}+\cdots +s^{m-n-1}\alpha^{m-1}d_{0}$$

$$+n\alpha^{n}+(n+1)\alpha^{n+1}+\cdots +(m-1)\alpha^{m-1}$$

$$\leq \alpha^{n}d_{0}(1+\alpha s+(\alpha s{)}^{2}+(\alpha s{)}^{3}+\cdots +s^{m-n-1}\alpha^{m-n-1})$$

$$+\sum_{i=n}^{m-1}is^{i-n}\alpha^{i}.$$

In the limiting case, when m,n→∞,


$$d(g_{n},g_{m})=0.$$

So {*g*_*n*_} is a Cauchy sequence in *G*. By completeness of *G*, there exists h∈G such that $g_{n}\to h$. Now we will prove that *h* is a fixed point of *S*.


$$d(h,Sh)\leq d(h,g_{n})+sd(g_{n},S(h)).$$

By Lemma 2.8 (1),


$$\leq d(h,g_{n})+sH(S^{2}(g_{n-1}),S^{2}(h))$$

$$\leq d(h,g_{n})+s[\alpha d(g_{n-1},h)+\beta d(S(g_{n-1}),(Sh))].$$

In the limiting case, when n→∞,


d(h,Sh)≤0,

d(h,Sh)=0.

Now *Sh* is closed and so h∈Sh. Hence, *h* is a fixed point of *S* as desired.

Here is a multivalued version of Theorem 2.2 for a convex contraction (see also [20],[24],[27]).

**Theorem 2.11:** Let (*G*, *d*) be a complete metric space and let S : G→CB(G) be a multivalued *F*-convex contraction satisfying:


$$2\tau +F(H(S^{2}(g),S^{2}(h))\leq F(a_{1}d(g,S(h))+a_{2}d(S(h),S^{2}(h))+b_{1}d(h,S(g))+$$

$$b_{2}d(S(g),S^{2}(g)))$$ (9)

for all g,h∈G, $a_{1},a_{2},b_{1},b_{2}\in (0,1)$ and $a_{1}+a_{2}+b_{1}+b_{2}\;<\;1$. Then *S* has a fixed point.

**Proof:** Let $g_{0}\in G$ be an arbitrary point of *G* and choose $g_{1}\in S(g_{0})$. If $g_{1}\in S(g_{1})$, then *g*_1_ is a fixed point of *S* and nothing to prove.

Assume that $g_{1}\notin S(g_{1})$. Then $S(g_{0})\neq S(g_{1})$.


$$2\tau +Fd(S^{2}(g_{0}),S^{3}(g_{0}))\leq 2\tau +F(H(S^{2}(g_{0}),S^{3}(g_{0}))+\tau$$

$$Fd(S^{2}(g_{0}),S^{3}(g_{0}))\leq F(H(S^{2}(g_{0}),S^{3}(g_{0})))+\tau$$

Let *g* = *g*_0_, *h* = *S*(*g*_0_) in (9) and set $K=\max\{d(x_{0},S(g_{0})),d(S(g_{0}),S^{2}(g_{0}))\}$,


$$F(H(S^{2}(g_{0}),S^{3}(g_{0})))$$

$$\leq F(a_{1}d(g_{0},S^{2}(g_{0}))+a_{2}d(S^{2}(g_{0}),S^{3}(g_{0}))+b_{1}d(S(g_{0}),S(g_{0}))$$

$$+b_{2}d(S(g_{0}),S^{2}(g_{0}))$$

$$\leq F(a_{1}d(g_{0},S^{2}(g_{0}))+a_{2}d(S^{2}(g_{0}),S^{3}(g_{0}))+b_{2}d(S(g_{0}),S^{2}(g_{0}))$$

$$\leq F(a_{1}d(g_{0},S(g_{0}))+a_{1}d(S(g_{0}),S^{2}(g_{0}))+b_{2}d(S(g_{0}),S^{2}(g_{0}))$$

$$+a_{2}H(S^{2}(g_{0}),S^{3}(g_{0}))$$

$$\leq F((a_{1}+a_{2}+b_{2})\max\{d(x_{0},S(g_{0})),d(S(g_{0}),S^{2}(g_{0}))\}+a_{2}H(S^{2}(g_{0}),S^{3}(g_{0}))$$

$$\leq F\left(\frac{2a_{1}+b_{2}}{1-a_{2}}K\right)$$ (10)

Since *F* is strictly increasing and τ is greater than zero, (10) becomes


$$\;<\;\frac{2a_{1}+b_{2}}{1-a_{2}}K.$$

$$\text{So}\;H(S^{2}(g_{0}),S^{3}(g_{0}))\leq \lambda K$$ (11)

where $\lambda =\frac{2a_{1}+b_{2}}{1-a_{2}}$. Substituting *g* = *S*(*g*_0_), $h=S^{2}(g_{0})$ in (9),we have


$$2\tau +Fd(S^{3}(g_{0}),S^{4}(g_{0}))$$

$$\leq 2\tau +F(H(S^{3}(g_{0}),S^{4}(g_{0}))+\tau$$

$$\leq F(a_{1}d(S(g_{0}),S^{3}(g_{0}))+a_{2}d(S^{3}(g_{0}),S^{4}(g_{0}))+b_{1}d(S^{2}(g_{0}),S^{2}(g_{0}))+$$

$$b_{2}d(S^{2}(g_{0}),S^{3}(g_{0}))$$

$$\leq F(a_{1}d(S(g_{0}),S^{2}(g_{0}))+a_{1}d(S^{2}(g_{0}),S^{3}(g_{0}))+b_{2}d(S^{2}(g_{0}),S^{3}(g_{0}))+$$

$$a_{2}H(S^{3}(g_{0}),S^{4}(g_{0}))$$

$$\leq F\left(\frac{2a_{1}+b_{2}}{1-a_{2}}K\right)$$

Given that *F* is strictly increasing and τ>0


$$\;<\;\frac{2a_{1}+b_{2}}{1-a_{2}}K$$

$$H(S^{3}(g_{0}),S^{4}(g_{0}))\leq \lambda K$$

Similarly, we can show that


$$H(S^{4}(g_{0}),S^{5}(g_{0}))\leq \lambda^{2}K$$

$$H(S^{5}(g_{0}),S^{6}(g_{0}))\leq \lambda^{3}K$$

By following this process, we obtain


$$H(S^{n}(g_{0}),S^{n+1}(g_{0}))\leq F{\left(\frac{2a_{1}+b_{2}}{1-a_{2}}\right)}^{n-2}K$$

As *F* is strictly increasing and τ is greater than zero, we have


$$H(S^{n}(g_{0}),S^{n+1}(g_{0}))\;<\;{\left(\frac{2a_{1}+b_{2}}{1-a_{2}}\right)}^{n-2}K$$

$$H(S^{n}(g_{0}),S^{n+1}(g_{0}))\leq \lambda^{n-2}K$$

For *m* > *n*, we need to show {*g*_*n*_} is a Cauchy sequence in *G*. Using the triangular inequality, we obtain


$$H(S^{n}(g_{0}),S^{m}(g_{0}))\leq H(S^{n}(g_{0}),S^{n+1}(g_{0}))+H(S^{n+1}(g_{0}),S^{n+2}(g_{0}))+\cdots +$$

$$H(S^{m-1}(g_{0}),S^{m}(g_{0}))$$

$$\leq \lambda^{n-2}K+\lambda^{n-1}+\cdots +\lambda^{m+n-3}K$$

$$\leq \left(\frac{\lambda^{n-1}}{1-\lambda }\right)K\;\text{(a geometric series converging to zero).}$$

$$\text{Hence}\;\lim_{n\to \infty }H(S^{m}(g_{0}),S^{n}(g_{0}))=0$$

This demonstrates that the sequence {*g*_*n*_} is Cauchy in *G*, implying the existence of an element z∈G such that


$$\lim_{n\to \infty }g_{n}=\lim_{n\to \infty }S^{n}g_{0}=z.$$

Now we have to prove that *z* is a fixed point of *S*. As *S* is orbitally continuous, so we get


$$z=\lim_{n\to \infty }S(S^{n}(g_{0}))\in Sz$$

This shows that *z* is a fixed point of *S*.

**Definition 2.12** Let (*G*, *d*) be a complete metric space. A mapping S : G→CB(G) is a weak convex contraction if it satisfies


$$H(S^{2}(g),S^{2}(h))$$

$$\leq a_{0}d(g,h)+b_{0}d(h,S(g))+a_{1}d(S(g),(Sh))+b_{1}d(S(h),S^{2}(g))$$

for all g,h∈G and $b_{0},b_{1}\geq 0$ and $0\;<\;a_{0},a_{1}\;<\;1$ and $a_{0}+a_{1}\;<\;1.$

Here is an extention of Theorem 3 of Berinde and Berinde [2] for a weak convex contraction.

**Theorem 2.13** Let (*G*, *d*) be a complete metric space and S:G→CB(G) a multivalued weak convex contraction. Then

(i) Fix(S)≠ϕ;
(ii) For every initial point $g_{0}\in G$, there exists a sequence $\{g_{n}{\}}_{n=0}^{\infty }$ generated by the operator *S* that converges to a fixed point *u* of *S*, for which the following estimates hold:
  $$d(g_{n},u)\leq \frac{h^{n}}{1-h}d(g_{0},g_{1}),n=0,1,2,...,$$ (12)
  $$d(g_{n},u)\leq \frac{h}{1-h}d(g_{n-1},g_{n}),n=0,1,2,...,$$ (13)
  for a certain real number *h* < 1.

**Proof:** (i) Let *q* > 1. Let $g_{0}\in G$ and $g_{1}\in Sg_{0}$. We consider two cases based on the Hausdorff distance between the iterates of *S*.

**Case 1:** If $H(S^{2}g_{0},S^{2}g_{1})=0$, then $S^{2}g_{0}=S^{2}g_{1}$.

Now $S^{2}(g_{0})=S(S(g_{0}))$ implies that $S(g_{0})=S(g_{1})$ (cf. Remark 1.3(i)). Therefore $g_{1}\in Sg_{1}$ gives Fix(S)≠ϕ and the proof is complete.

**Case 2:** Let $H(S^{2}g_{0},S^{2}g_{1})\neq 0$. By Lemma 1.28, there exists $g_{2}\in Sg_{1}$ such that $d(g_{1},g_{2})\leq qH(S^{2}g_{0},S^{2}g_{1})$.


$$\leq q[a_{0}d(g_{0},g_{1})+b_{0}d(g_{1},Sg_{0})+a_{1}d(Sg_{0},Sg_{1})+b_{1}d(Sg_{1},S^{2}g_{0})]$$

$$\leq q[a_{0}d(g_{0},g_{1})+b_{0}d(g_{1},g_{1})+a_{1}d(g_{1},g_{2})+b_{1}d(g_{2},g_{2})]$$

$$\leq \frac{q}{\left(1-a_{1}\right)}a_{0}d(g_{0},g_{1})$$

$$\leq q_{1}a_{0}d(g_{0},g_{1})\;\;\text{where}\;q_{1}=\frac{q}{\left(1-a_{1}\right)}$$

We take $q_{1}\;>\;1$ such that


$$h=q_{1}a_{0}\;<\;1.$$

Hence


$$d(g_{1},g_{2})\;<\;hd(g_{0},g_{1}).$$

If $H(S^{2}g_{1},S^{2}g_{2})=0$, then $S(S(g_{1}))=S(S(g_{2}))$, i.e., $g_{2}\in Sg_{2}$.

Assume that $H(S^{2}g_{1},S^{2}g_{2})\neq 0$. Again by Lemma 1.28, there exists $g_{3}\in Sg_{2}$ such that


$$d(g_{2},g_{3})\leq hd(g_{1},g_{2}).$$

In this way, we construct an orbit $\{g_{n}{\}}_{n=0}^{\infty }$ at *g*_0_ for *S* satisfying

$$d(g_{n},g_{n+1})\leq hd(g_{n-1},g_{n}),\;n=1,2,...$$ (14)

By (14), we obtain inductively

$$d(g_{n},g_{n+1})\leq h^{n}d(g_{0},g_{1}).$$ (15)

Hence

$$d(g_{n+k},g_{n+k+1})\leq h^{k+1}d(g_{n-1},g_{n}),\;k\in N,n\geq 1.$$ (16)

Similarly, by (15), we have

$$d(g_{n},g_{n+p})\leq \frac{h^{n}(1-h^{p})}{1-h}d(g_{0},g_{1}),n,p\in N,$$ (17)

Considering 0<h<1, it can be concluded that the sequence $\{g_{n}{\}}_{n=0}^{\infty }$ constitutes a Cauchy sequence. Given that (G,d) is a complete metric space, it follows that the sequence $\{g_{n}{\}}_{n=0}^{\infty }$ converges. Let

$$u=\lim_{n\to \infty }g_{n}.\;\;\;\;$$ (18)

Then by (*) in the proof of Lemma 1.28, we get


$$d(u,Su)\leq d(u,g_{n+1})+d(g_{n+1},Su)$$

$$\leq d(u,g_{n+1})+H(S^{2}g_{n},S^{2}u)$$

$$\leq d(u,g_{n+1})+[a_{0}d(g_{n},u)+b_{0}d(u,Sg_{n})+$$

$$a_{1}d(Sg_{n},Su)+b_{1}d(Su,S^{2}g_{n})]$$ (19)

Letting n→∞ in (19) and using the fact that $g_{n+1}\in Sg_{n}$ imply by (18) that $d(u,Sg_{n})\to 0$, as n→∞. So we get


d(u,Su)=0

As *Su* is closed, so u∈Su.

(ii) Let p→∞ in (17). Then ${\text{h}}^{\text{p}}$ approaches 0 and so we get


$$d(g_{n},u)\leq \frac{h^{n}}{1-h}d(g_{0},g_{1}).$$

This proves (12).


$$d(g_{n+k},g_{n+k+1})\leq h^{k+1}d(g_{n-1},g_{n}),\;k\in N.\;$$

In the same way when k→∞ in (16), we get

$$d(g_{n},u)\leq \frac{h^{n}}{1-h}d(g_{0},g_{1}).$$ (20)

At the end of this section, we present relation among various concepts, used in this work in the form of a diagram:

**1- Contraction**

**2- Convex Contraction**

**3- Hardy and Rogers Convex Contraction**

**4- Weak Convex Contraction**

**5- Generalized Convex Contraction**

**6- F-Contraction**

### 2.3 Diagram

Here the arrow stands for the inclusion.



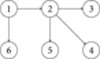



## 3 Application

Let G=C[a,b] represent the vector space of all continuous real-valued functions on [*a*, *b*] endowed with the usual metric. Then (*G*, *d*) is a complete metric space.

The non-linear Fredholm integral equation is given as follows:

$$h(t)=v(t)+\frac{1}{b-a}\int_{a}^{b}k(t,s,h(s))\;ds$$ (21)

where t,s∈[a,b], k:[a,b]×[a,b]×G→ℝ and v:[a,b]→ℝ are continuous and *v*(*t*) is a given function in *G*.

The solution of non-linear Fredhalm integral equation has been obtained for Hardy and Rogers convex contraction in [9]. We apply our Theorem 2.2 to solve non-linear Fredhalm integral equation for a Chatterjea convex contraction.

**Theorem 3.1:** Let G=C[a,b] be the equipped with the usual metric.

Assume that (i) S:G→G is given by

$$Sg(t)=v(t)+\frac{1}{b-a}\int_{a}^{b}k(t,s,g(s))\;ds$$ (22)

(ii) For $a_{1},a_{2},b_{1},b_{2}\in [0,1)$, g,h∈G with g≠h and s,t∈[a,b], we have


$$\left|k(t,s,S(g))-k(t,s,S(h))\right|\leq a_{1}d(g(s),S(h(s)))+a_{2}d(S(h(s)),S^{2}\;h(s)$$

$$+b_{1}d(h(s),S(g(s)))+b_{2}d(S(g(s)),S^{2}\;g(s))$$ (23)

If *S* is orbitally continuous, the integral operator defined by equation (22) possesses a unique solution z∈G. Moreover, for any initial value $g_{0}\in G$, it holds that $S(g)\neq g_{n}$ for all n∈ℕ∪{0}. Consequently, we have $\lim_{n\to \infty }S^{n}(g_{0})=z.$

**Proof**

Let $g_{0}\in G$ be an arbitrary point. Define a sequence {*g*_*n*_} in *G* by $S(g_{n})=S^{\left(n+1\right)}(g_{0})$ for all n≥0. By (22), we have,


$$g_{n+1}=S(g_{n}(t))=v(t)+\frac{1}{b-a}\int_{a}^{b}k(t,s,g_{n}(s))\;ds$$

We must demonstrate that *S* is a Chatterjea two sided convex contraction on *C*[*a*, *b*]. Use of (22) and (23) yields


$$\left|S^{2}\;g(t)-S^{2}\;h(t)\right|$$

$$=\frac{1}{\left|a-b\right|}\left|\int_{a}^{b}k(t,s,Sg(g))ds-\int_{a}^{b}k(t,s,Sh(s))ds\right|$$

$$\leq \frac{1}{b-a}\int_{a}^{b}\left\{|k(t,s,Sg(s))-k(t,s,Sh(s))|\right\}ds$$

$$\leq \frac{1}{\left|b-a\right|}\int_{a}^{b}\{|(a_{1}|g(s)-Sh(s)|+a_{2}|Sh(s)-S^{2}\;h(s)|+$$

$$b_{1}|h(s)-Sg(s)|+b_{2}|Sg(s)-S^{2}\;g(s)|)|\}ds$$

$$\leq \frac{\left(a_{1}+a_{2}+b_{1}+b_{2}\right)}{\left|b-a\right|}\int_{a}^{b}\max\{|g(s)-Sh(s)|,|Sh(s)-S^{2}\;h(s)|,$$

$$|h(s)-Sg(s)|,|Sg(s)-S^{2}\;g(s)|\}ds$$

$$\leq \frac{k}{b-a}\max\{|g(s)-Sh(s)|,|Sh(s)-S^{2}\;h(s)|,$$

$$|h(s)-Sg(s)|,|Sg(s)-S^{2}\;g(s)|\}\int_{a}^{b}ds$$

$$\leq k\max\left\{d(g,S(h)),d(S(h),S^{2}(h)),d(h,S(g)),d(S(g),S^{2}(g))\right\}$$

where $k=a_{1}+a_{2}+b_{1}+b_{2}\;<\;1$ for all g,h∈G with g≠h.

Since *G* is a complete metric space, therefore the iterative process converges to a specific point z∈G (i.e. $\lim_{n\to \infty }g_{n}=z$). By orbital continuity of *S*, we can establish that *z* is a fixed point of *S*. Thus all the conditions of Theorem 2.2 are satisfied and so by it’s conclusion the non-linear Fredholm integral operator *S* defined by (22) has a unique solution.

Now we provide an example to demonstrate the application of Theorem 3.1

**Example 3.2:**

Let us consider the operator S : C[0,1]→C[0,1] defined as:

$$Sg(t)=\frac{1}{2}g(t)+\int_{0}^{1}\left(\frac{1}{3}g(s)+\frac{1}{4}(t+s)\right)ds,$$ (24)

where g∈C[0,1], and t,s∈[0,1]. The kernel k(t,s,g(s)) is given by:


$$k(t,s,g(s))=\frac{1}{3}g(s)+\frac{1}{4}(t+s).$$

Let us choose the following constants:


$$a_{1}=\frac{1}{5},\;a_{2}=\frac{1}{6},\;b_{1}=\frac{1}{7},\;b_{2}=\frac{1}{8},$$

which satisfy the requirement:


$$a_{1}+a_{2}+b_{1}+b_{2}=\frac{1}{5}+\frac{1}{6}+\frac{1}{7}+\frac{1}{8}\;<\;1.$$

Let g,h∈C[0,1]. We must demonstrate that:


$$|k(t,s,S(g))-k(t,s,S(h))|\leq a_{1}|g(s)-S(h(s))|+a_{2}|S(h(s))-S^{2}(h(s))|+$$

$$b_{1}|h(s)-S(g(s))|+b_{2}|S(g(s))-S^{2}(g(s))|.$$

Substituting the kernel $k(t,s,g(s))=\frac{1}{3}g(s)+\frac{1}{4}(t+s)$, we get:


$$|k(t,s,S(g))-k(t,s,S(h))|=\left|\frac{1}{3}S(g(s))-\frac{1}{3}S(h(s))\right|.$$

Substituting the expressions for S(g(s)) and S(h(s)), we have:


$$\left|\frac{1}{3}\left(\frac{1}{2}g(s)+\int_{0}^{1}k(t,u,g(u))du\right)-\frac{1}{3}\left(\frac{1}{2}h(s)+\int_{0}^{1}k(t,u,h(u))du\right)\right|.$$

By the given nature of *g* and *h*, the subsequent inequality holds:


$$|k(t,s,S(g))-k(t,s,S(h))|\leq a_{1}|g(s)-S(h(s))|+a_{2}|S(h(s))-S^{2}(h(s))|+$$

$$b_{1}|h(s)-S(g(s))|+b_{2}|S(g(s))-S^{2}(g(s))|.$$

So *S* satisfies (23). The operator *S* being continuous is orbitally continuous.

Thus, all the requirements of Theorem 3.1 are satisfied and so the operator *S* defined by (24) has a unique solution.

## 4 Conclusion

In this work, we have proved fixed point theorems for single-valued convex contraction mappings in b-metric spaces. Generalizing, some of these results for multivalued convex contraction mappings, an analogue of well-known theorems of Nadler and Istratescu are obtained. A result for an F-convex contraction is also established. A diagram is included here to provide an insight for the relationship among various convex contractions. A special case of Theorem 2.11 is applied to solve a non-linear Fredholm integral equation in the context of a Chatterjea two-sided convex contraction.

## 5 Open problems

Establish Theorems 2.2, 2.5, 2.10 and 2.13 for common fixed points and coincidences of convex contractions.
