# Supplementary material for: Fixed points of multivalued convex contractions with application
Source: PLoS One. 2025 May 12;20(5):e0321860. doi: 10.1371/journal.pone.0321860 (PMC12068645; doi:10.1371/journal.pone.0321860)
Supplement: Attachment — Submitted filename: Report PLOS ONE.pdf [file pone.0321860.s001.pdf]

Referee Report on the Manuscript

Fixed Points of Multivalued Convex Contractions with Application

Abdul Rahim Khan, Hamed H. Al-Sulami, Muhammad Rashid, Faiza Shabbir

The authors have established fixed point results for single-valued convex contraction-type mappings in b-metric spaces and extended some of their results for multivalued convex contractions and F-convex contractions. In this manuscript, equivalents of the well-known theorems of Nadler and Istratescu are demonstrated for multivalued convex contractions. The new result established in Theorem 2.11 is applied to solve a non-linear Fredholm integral equation in the context of a Chatterjea two-sided convex contraction.

The results obtained in this article are of fundamental nature and their proof seems correct and logical. The paper is well written, and the use of terminology and symbols is consistent. In my view this work lays the foundation for more investigation in this important area of mathematics. I strongly recommend the publication of this paper in PLOS ONE.
