# Supplementary material for: Fixed points of multivalued convex contractions with application
Source: PLoS One. 2025 May 12;20(5):e0321860. doi: 10.1371/journal.pone.0321860 (PMC12068645; doi:10.1371/journal.pone.0321860)
Supplement: Attachment — Submitted filename: Response to Reviewr_054643.docx [file pone.0321860.s002.docx]

**Rebuttal Letter**
**Manuscript ID:** PONE-D-24-59912
**Title:** *Fixed Points of Multivalued Convex Contractions with Application*
**Journal:** *PLOS ONE*

**Dear Editors,**

We sincerely appreciate the reviewers' insightful comments and valuable suggestions. Below, we provide our point-by-point responses along with the corresponding revisions made to the manuscript.

**Reviewer #1 Comments and Responses**

**1. Comment:** On page 1, under Definition 1.1, add *"for any g,h∈Gg,h \in Gg,h∈G"* for clarity.
**Response:** We believe the reviewer intended to refer to *Definition 1.2* rather than *Definition 1.1*. Accordingly, we have added *"for any g,h∈Gg,h \in Gg,h∈G"* in *Definition 1.2* to enhance clarity.

**2. Comment:** On pages 17 and 18, replace ∅\varnothing∅ with ∅\emptyset∅.
**Response:** In the updated PLOS ONE template, the relevant section now appears on *page 13*. We have replaced all occurrences of ∅\varnothing∅ with ∅\emptyset∅ to maintain consistency in notation.

**3. Comment:** On page 20, lines 11 and 13 from the bottom should be combined with line 20 from the bottom.
**Response:** In the revised PLOS ONE template, this section now appears on *page 14*. We have merged the mentioned lines to improve readability and coherence.

**4. Comment:** Check the page numbers of references [9] and [26].
**Response:**

- **Reference [9]:** The issue notation has been updated to *6(1)*.
- **Reference [26]:** The missing volume number has been added.

**Final Remarks**

All suggested revisions have been carefully implemented. We sincerely appreciate the reviewers’ constructive feedback, which has contributed to improving the manuscript. We kindly request your consideration of our revised submission.

**Best regards,**
Abdul Rahim Khan
abdulrahimkhan@isp.edu.pk
